# Striatal dopamine D2-like receptors availability in obesity and its modulation by bariatric surgery: a systematic review and meta-analysis

**DOI:** 10.1038/s41598-023-31250-2

**Published:** 2023-03-27

**Authors:** Gabriela Ribeiro, Ana Maia, Gonçalo Cotovio, Francisco P. M. Oliveira, Durval C. Costa, Albino J. Oliveira-Maia

**Affiliations:** 1 Champalimaud Research and Clinical Centre, Champalimaud Foundation, Av. de Brasília, Doca de Pedrouços, 1400-038 Lisboa, Portugal; 2 Lisbon Academic Medical Centre PhD Program, Faculdade de Medicina da Universidade de Lisboa, Avenida Professor Egas Moniz, 1649-028 Lisboa, Portugal; 3 Nova Medical School, Faculdade de Ciências Médicas, NMS, FCM, Universidade Nova de Lisboa, Campo Mártires da Pátria 130, 1169-056 Lisboa, Portugal; 4 Department of Psychiatry and Mental Health, Centro Hospitalar de Lisboa Ocidental, Rua da Junqueira, 126, 1340-019 Lisboa, Portugal; 5 Present Address: Nutrition and Metabolism Department, Nova Medical School, Faculdade de Ciências Médicas, NMS, FCM, Universidade Nova de Lisboa, Campo Mártires da Pátria 130, 1169-056 Lisboa, Portugal

**Keywords:** Feeding behaviour, Motivation, Reward

## Abstract

There is significant evidence linking a ‘reward deficiency syndrome’ (RDS), comprising decreased availability of striatal dopamine D2-like receptors (DD2lR) and addiction-like behaviors underlying substance use disorders and obesity. Regarding obesity, a systematic review of the literature with a meta-analysis of such data is lacking. Following a systematic review of the literature, we performed random-effects meta-analyses to determine group differences in case–control studies comparing DD2lR between individuals with obesity and non-obese controls and prospective studies of pre- to post-bariatric surgery DD2lR changes. Cohen's *d* was used to measure effect size. Additionally, we explored factors potentially associated with group differences in DD2lR availability, such as obesity severity, using univariate meta-regression. In a meta-analysis including positron emission tomography (PET) and single-photon emission computed tomography (SPECT) studies, striatal DD2lR availability did not significantly differ between obesity and controls. However, in studies comprising patients with class III obesity or higher, group differences were significant, favoring lower DD2lR availability in the obesity group. This effect of obesity severity was corroborated by meta-regressions showing inverse associations between the body mass index (BMI) of the obesity group and DD2lR availability. Post-bariatric changes in DD2lR availability were not found, although a limited number of studies were included in this meta-analysis. These results support lower DD2lR in higher classes of obesity which is a more targeted population to explore unanswered questions regarding the RDS.

## Introduction

Obesity is a global challenge for public health, with well-known associations with morbidity and mortality^1,2^. One major concern regarding obesity treatment is poor sustainability of lifestyle modifications over time^2,3^. On the other side, bariatric surgery, namely gastric bypass and sleeve gastrectomy, while indicated for severe obesity only, is a successful intervention for sustained weight loss^4–6^. Furthermore, some anti-obesity drugs, namely phentermine/topiramate, naltrexone/bupropion, liraglutide and orlistat, are used as co-adjuvants of behavioral modification^7^. The first two options mentioned above include drugs acting, at least in part, via the dopaminergic system^8^, suggesting potential clinical relevance of this neurotransmitter system in obesity.

Indeed, the importance of the brain in feeding behavior regulation is well established^9–12^. Several brain structures take part in this function^12^. For example, the hypothalamus integrates appetite-regulating signals, while the caudal brainstem coordinates ingestion, digestion, and food absorption^12^. Reward and learning processes relevant to feeding behavior occur in corticolimbic regions^12^, with the striatum, including the caudate, putamen and nucleus accumbens (NAc), as a crucial structure for the processing of food reward^13,14^. Neurons in the striatum can express the five types of dopamine receptors, with D1 and D2 receptors being predominant^13^.

The striatum's decreased availability of dopamine D2-like receptors (DD2lR) has been reported in patients with drug misuse syndromes^9,10,15,16^. Comparable findings were found in severe obesity, with reports of decreased DD2lR availability and a negative correlation between DD2lR availability and body mass index (BMI)^17^, contributing to converging theories for excessive eating and substance misuse^9–11^. According to these theories, a blunted reward system, resulting, for example, from reduced DD2lR availability, is suggested to enhance the drive to overeat as a compensation operating through increased dopamine release^9–11^. On the other hand, DD2lR availability may decrease due to chronic dopaminergic overstimulation resulting from overeating^9–11^. These interpretations align with the concept of a 'reward deficiency syndrome', integrating reduced DD2lR availability with addiction-like behaviors^15,16,18–20^.

In humans, positron emission tomography (PET) and single-photon emission computed tomography (SPECT) are the primary techniques to assess DD2lR availability, using several radiopharmaceuticals with an affinity for DD2lR^12,21,22^. While some PET and SPECT studies compared individuals with obesity and controls for DD2lR availability^12^, only a few studies performed such assessments before and after bariatric surgery^12^. However, results are not consensual, with reports of decreased, increased or unaltered DD2lR availability in obesity, and only one meta-analysis, restricted to [^11^C]raclopride PET studies published until 2015^23^. Although previous non-systematic reviews raised interesting points to conciliate discrepancies^12,22,24^, for guidance of future studies a systematic review and meta-analysis is crucial. Furthermore, literature on prospective assessment of striatal DD2lR in bariatric surgery has not been systematically reviewed or meta-analyzed. Therefore, with this work, we aimed to systematically review the literature for quantitative evidence that DD2lR availability is lower in obesity than in non-obese controls and increases after bariatric surgery.

## Material and methods

### Search strategy

Before starting the study, the proposed methodology was published in the Prospero Platform (registration number PROSPERO 2020: CRD42021229282). We systematically reviewed the literature to identify case–control studies comparing DD2lR between adults with obesity [body mass index (BMI) ≥ 30 kg/m^2^] and non-obese controls (BMI < 30 kg/m^2^). We also systematically reviewed prospective studies that assessed changes in DD2lR availability following bariatric surgery, either gastric bypass or sleeve gastrectomy. The literature search was performed using a predetermined syntax based on Medical Subject Headings (MeSHs) terms. Search terms were defined according to the PRISMA statement, comprising the following fields: (1) nuclear medicine imaging techniques; (2) striatal DD2lR availability; (3) the disorder of interest—obesity; (4) the intervention of interest—bariatric surgery. In addition, we applied a filter to restrict search results to papers with human subjects, with no restrictions regarding publication year or country of origin (Supplementary Table 1). The search syntax was the following: (‘Positron Emission Tomography’ OR ‘PET’ OR ‘Single Photon Emission Computed Tomography’ OR ‘SPECT’) AND (‘dopamine’ OR ‘striatum’ OR ‘basal ganglia’ OR ‘putamen’ OR ‘caudate’ OR ‘accumbens’ OR ‘D2’ OR ‘DD2R’) AND (‘obesity’ OR ‘overweight’ OR ‘body mass index’ OR ‘BMI’ OR ‘bariatric’ OR ‘gastric bypass’ OR ‘sleeve’ OR ‘gastrectomy’). The standardized search was performed on three electronic bibliographic databases (PubMed, Web of Science, and Embase) on January 13, 2021.

### Study selection and risk of bias assessment

Duplicate articles were eliminated using Zotero 5.0.96.2, after which two researchers (GR and AM) performed article selection independently in three subsequent steps: title, abstract, and full-text review. Upon completing each step, the two researchers reached a consensus on articles selected for the following stage, with a third researcher participating if necessary. At the end of the full-text review, we manually searched the reference lists of the selected papers to identify additional relevant articles for the final complete paper consensus. Studies were selected according to the presence of both of the following inclusion criteria: (1) case–control studies, with cases defined as having obesity (BMI ≥ 30 kg/m^2^) and a control group without obesity (BMI < 30 kg/m^2^); or prospective cohort studies including patients with obesity undergoing bariatric surgery, either gastric bypass and sleeve gastrectomy, assessed before and after surgery; (2) assessment of DD2lR-specific binding expressed as the binding potential (BP) obtained from the region of interest (ROI)-based analyses. While we did not impose publication date, country of origin, or radiopharmaceuticals restrictions, we only considered English, French, Portuguese, Spanish or German-written articles and excluded animal studies and literature reviews.

Two researchers (GR and AM) independently assessed the methodological quality of each article included in the review by using the Newcastle–Ottawa scale (NOS)^25^, either for case–control or cohort studies. The final appraisal was reached by consensus. For this review, studies awarded a NOS score equal to or higher than eight were considered high-quality studies^26^.

### Data extraction

Data was extracted manually and independently by two researchers (GR and AM), including (1) striatal DD2lR BP (primary outcome) and putamen, caudate, and NAc DD2lR BP (secondary outcomes); (2) first author name, publication title, country of origin, year, journal, study type; (3) sample size, percentage of men, mean age at inclusion, years of education, BMI at inclusion, BMI after surgery and percent weight loss (in the case of prospective cohort studies); (4) nuclear medicine methods namely scanner type, radiopharmaceuticals, co-registration with magnetic resonance imaging (MRI), brain reference region used in ROI analyses and acquisition time; (5) percentage of smokers, current or past major psychiatric disorders (including eating disorders, alcohol or substance abuse), severe medical conditions (including neurological conditions and diabetes mellitus type 1 or 2), history of head trauma, current or past exposure to dopaminergic or anti-diabetic medication, pregnancy or breastfeeding. We also contacted the authors of 16 papers to obtain additional information about study eligibility or DD2lR availability that was not reported in the papers. The requested information was provided for ten articles, including four databases with individual socio-demographic, BMI, and DD2lR data^27–30^. Although two studies had overlapping cohorts^28,29^, the corresponding author provided non-overlapping individual data. Some papers did not report DD2lR availability for the whole striatum but instead estimated specific ROIs (putamen, caudate, and Nac/ventral striatum). Thus, to maximize the number of studies included in the meta-analysis, data were either directly extracted from articles^31–33^ or analyzed using databases provided by the authors^27–30^. The research team determined the weighting factors for each region based on volumes obtained from the international consortium for brain mapping (ICBM) Montreal neurological institute (MNI) template^34^, resulting in the following formula:

$$Striatal DD2lRBP = (0.54 \times Putamen BP) + (0.42 \times Caudate BP) + (0.04 \times NAc/VS BP).$$

In cases where we calculated the weighted mean with values extracted from the papers, rather than databases^31–33^ the formula to calculate the BP standard deviation (SD) was the following: *SD* = *weighted mean* × *coefficient of variation (CoV) of the remaining studies (where CoV* = *σ∕μ)*. For Guo J et al.^31^, however, that reported mean and 95% confidence intervals (CI), SD was calculated using the following formula^35^: *SD* = *√N* × *(upper limit − lower limit) ∕ (2* × *value from t-distribution)*. In Steele KE et al.^36^, DD2lR BP was obtained from a graph using a millimetric grid. Also, in this study^36^, we calculated the SD of demographic data using individual mean values reported in the paper.

### Statistical analysis

Considering the anticipated high heterogeneity between studies, we performed a random-effects meta-analysis to compare striatal DD2lR availability between individuals with obesity and non-obese controls. The model computed the between-groups effect size (Cohen's *d*) weighted by sample size and I-squared (I^2^) index to assess the heterogeneity of effect sizes. In addition, when at least ten studies were available^35^, we tested publication bias using Egger's test^37,38^, visual inspection of funnel plot^38,39^, and leave-one-out meta-analyses. Finally, to address the hypothesis that bariatric surgery induces changes in striatal DD2lR availability, we used random-effects meta-analysis, computing the post-surgical change in striatal DD2lR BP. The effect size of post-surgery change (Cohen's *d*) was calculated according to Borenstein formulas for correlated data^40^, assuming an *r* of 0.5, according to prior literature^40–42^.

Subgroup meta-analyses were performed by comparing groups of papers according to variables of interest: (1) obesity severity—class I-II (BMI 30 to < 40 kg/m^2^) and class III or higher (BMI ≥ 40 kg/m^2^); (2) ROIs—putamen, caudate and NAc/ventral striatum; (3) scanner type—PET and SPECT; (4) radiopharmaceuticals—[^11^C]raclopride, [^123^I]IBZM, [^18^F]fallypride and [^11^C]NMB (we did not analyze [^11^C]PHNO since there was only one study); (5) MRI co-registration—PET with co-registration and PET without co-registration; (6) Study quality according to NOS score—high quality (≥ 8) and others (< 8)^26^. We also performed a univariate meta-regression to test the association between obesity level and DD2lR availability, using the Cohen's *d* of striatal DD2lR BP (obesity vs. controls) as the dependent variable. The independent variables were the mean BMI of each group or the BMI difference between cases and controls (ΔBMI). This analysis included 11 studies, following best practices for meta-regression^35^. In addition, we performed similar metaregression with age or gender as independent variables.

Descriptive statistics and weighted means were computed using SPSS version 25 (SPSS Inc., Chicago, IL, USA) and all meta-analyses and meta-regressions using Stata Statistical Software: Release 15 (StataCorp LLC, College Station, TX). Finally, figures and graphs were prepared using Adobe Illustrator version CC 2019 (Adobe Inc., San Jose, CA, USA).

## Results

### Search results

**Figure 1:**
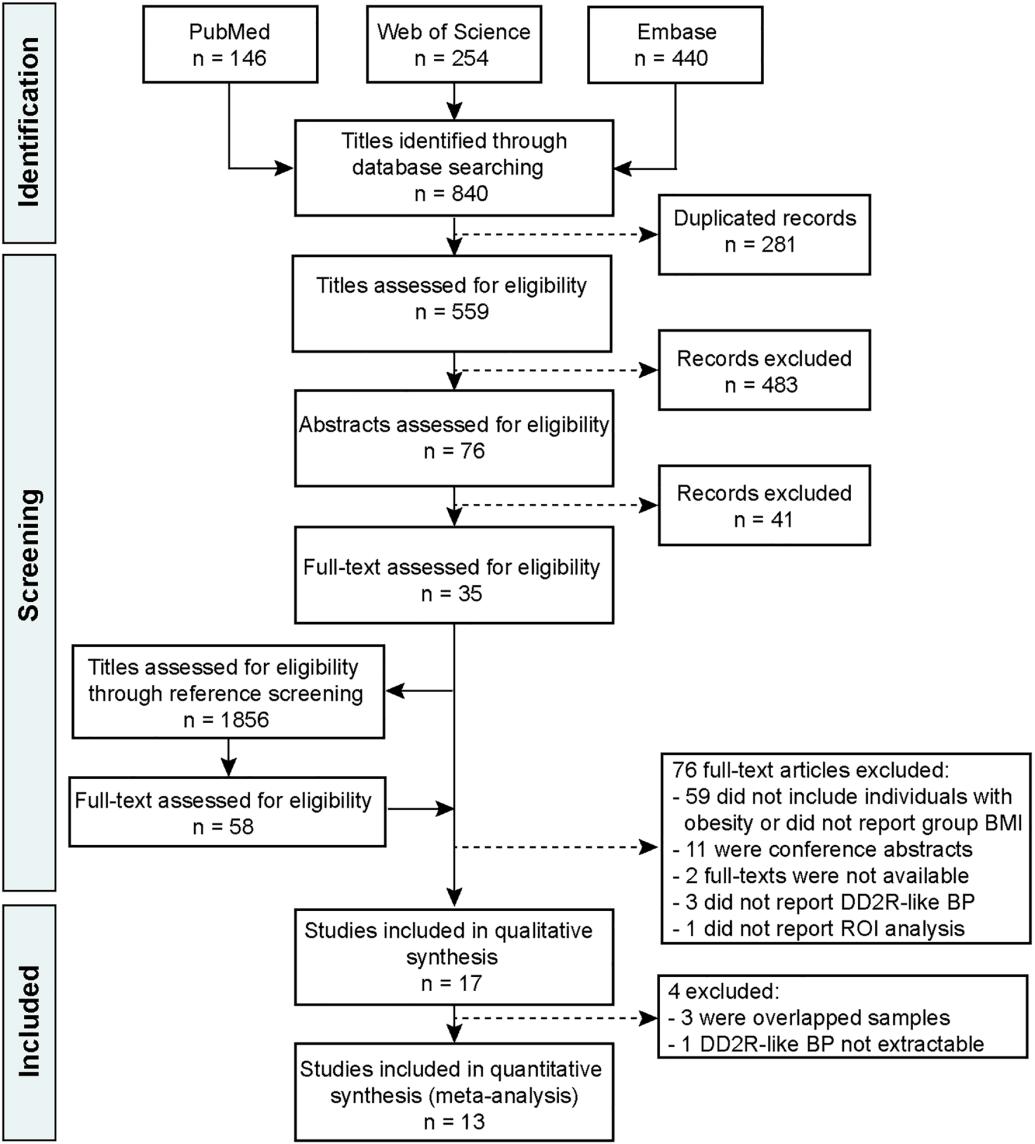
Article selection flowchart. We performed a systematic review according to PRISMA guidelines, and from an initial pool of 840 titles, 17 articles were eligible for qualitative analysis, of which 13 were included in the meta-analysis. These included ten case–control studies comprising obesity and control groups and three prospective cohort studies of patients with obesity assessed before and after bariatric surgery. Of the prospective studies, one study also comprised a control group at baseline, and thus the data was included in case–control studies. Abbreviations: DD2lR—striatal dopamine D2-like receptor; BP—Binding Potential; ROI—Region of Interest.

From an initial pool of 559 articles, 17 articles^17,23,27–33,36,43–49^ were eligible for systematic review (see Fig. 1 for the article selection flowchart). The 17 studies provided 13 case–control comparisons between individuals with obesity and controls and 4 were prospective cohort studies conducted before and after bariatric surgery. Three of the studies had overlapping cohorts that could not be adequately resolved^43,46,48^, and thus the overlapping longitudinal article with shorter follow-up was excluded from the meta-analyses^48^. Regarding case–control studies, we retained those with the primary aim of comparing striatal DD2lR BP between obesity and control groups over studies that addressed other questions^43,46^. Furthermore, we could not extract striatal DD2lR BP values from one article^23^, and thus it was also excluded from meta-analyses.

### Systematic review

Regarding case–control studies, sample sizes were mostly small to moderate, with a median sample size of 15 participants in both groups, ranging from 6 to 27 in the obesity group and 10 to 112 in the controls. Prospective cohort studies also had small samples, with a median of 8 participants. In case–control studies groups had, on average, similar age and gender distribution, but with some studies not including male participants (Table 1 summarizes the characteristics of the included studies). However, the BMI of the obesity group was heterogeneous across studies, with seven comprising individuals with class III obesity or higher^17,23,28,43–46^, and the remaining six^27,29–32,47^ including class I or class II obesity. Regarding prospective cohort studies, all samples had class III obesity and the follow-up time was mainly short (6–9 weeks)^33,36,48^, with only one study^49^ reporting long-term assessment (2.1 to 3.6 years). Three of four studies^36,48,49^ comprised gastric bypass patients only, with the remaining prospective study^33^ including patients after gastric bypass or sleeve gastrectomy. Most studies (12 [70.6%]) were conducted in the United States, with only a minority (5 [29.4%]) comprising European participants from The Netherlands (4 [23.5%]) and Finland (1 [5.9%]). The majority of the studies (13 [76.5%]) used PET^17,23,27–33,36,43,46,47^ with different radiopharmaceuticals, namely [^11^C]raclopride^17,23,27,33,36,43^, [^11^C]NMB^28,29,46,47^, [^18^F]fallypride^30,31^, and [^11^C]PHNO^32^, while four studies (23.5%) used [^123^I]IBZM—SPECT^44,45,48,49^ (see Supplementary Table 2 for a summary of methodological characteristics). As assessed by the NOS, the average quality of the 17 studies was 6.9 (see Supplementary Table 3 for details).

**Table 1:** Description of eligible studies.

Publication	Number of subjects		Age at inclusion Years; Mean (SD)		Gender (% male)		Body mass index (kg/m^2^)		Striatal DD2lR BP		NOS
	Obesity	Controls	Obesity	Controls	Obesity	Controls	Obesity	Controls	Obesity vs. Controls	Post-op Changes	
**Case–control studies**											
Wang et al.^17^	10	10	38.9 (7.3)	37.5 (5.9)	50	70	51.2 (4.8)	24.7 (2.6)	↓		5
Volkow et al.^**a**^^43^	10	12	35.9 (10)	33.2 (8)	50	50	51 (5)	25 (3)	↓		5
de Weijer et al.^44^	15	15	37.8 (7)	28 (10.4)	0	0	46.8 (6.5)	21.7 (2.1)	↓		6
van De Giessen et al.^45^	15	15	36.3 (4)	38.5 (5.6)	0	0	42.9 (4.9)	21.8 (1.8)	↓		7
Karlsson et al.^23^	13	14	39.1 (10.7)	44.9 (12.9)	0	0	41.9 (3.9)	22.7 (2.9)	↔		7
Pepino et al.^**b**^^46^	22	19	31.2 (6.3)	28.3 (5.4)	13.6	21.1	40.3 (5)	22.5 (2.4)	↔		7
Eisenstein et al.^28^	15	15	32.5 (5.9)	29.7 (5.6)	20	26.7	40.3 (4.9)	22.6 (2.2)	↔ ^**d**^		7
Eisenstein et al.^29^	27	20	31.5 (6.6)	28.6 (5.2)	14.8	25	39.9 (4.8)	22.4 (2.4)	↔ ^**d**^		7
Eisenstein et al.^47^	22	17	31.4 (6.3)	28.5 (5.5)	13.6	23.5	39.6 (5.2)	22.1 (2)	↔		7
Guo et al.^31^	20	23	35.0 (7.4)	28.0 (6.1)	50	52	36.1 (4.6)	22.4 (2.5)	↑		6
Gaiser et al.^32^	14	14	37 (10.1)	34.9 (10.2)	71.4	71.4	35.3 (4.5)	22.3 (1.8)	↑		8
Dang et al.^30^	18	112	46.8 (18.1)	33.9 (17.6)	44.4	44.6	34.5 (4)	24.1 (3)	↔ ^**d**^		7
Wang et al.^27^	6	13	53.3 (3.4)	51.5 (3.2)	50	61.5	33 (2.4)	24.6 (2.8)	↔ ^**d**^		4
**Prospective cohort studies**											
de Weijer et al.^**c**^^48^	18		40.4 (8)		0		45.7 (6.3)			↔	9
van der Zwaal et al.^49^	11	11	44.3 (6)	40.5 (4)	0	0	45.2 (6.7)	21.9 (2)	↓	↑	9
Steele et al.^36^	5		32.2 (7.3)		0		45.2 (5.9)			↑	9
Dunn JP et al.^33^	5		45.8 (3.8)		0		43.2 (5.6)			↓	8

*Note*: Studies are organized by the body mass index (BMI) level of the obesity group.

Abbreviations: Striatal DD2lR BP, Striatal dopamine D2-like receptor binding potential (BP). NOS, Newcastle–Ottawa scale.

^a^Sample overlapped with Wang G-J et al., 2001.

^b^Sample overlapped with Eisenstein SA et al., 2013, Eisenstein SA et al., 2015a or Eisenstein SA et al., 2015b.

^c^Sample overlapped with van der Zwaal EM et al., 2016.

^d^DD2lR BP group values were calculated for metanalysis based on data provided by the authors.

In a qualitative analysis of the eligible case–control studies (n = 14), 5 (35.7%)^17,43–45,49^ reported decreased striatal dopamine D2 receptor availability in obesity relative to controls. All 5 of these studies included obesity groups with class III obesity and were conducted with [^11^C]raclopride—PET^17,43^ or [^123^I]IBZM—SPECT^44,45,49^. Three of these 5 studies had exclusively female participants^44,45,49^. Seven studies (50%)^23,27–30,46,47^ showed unaltered DD2lR availability between groups, one of which was not considered for analysis^46^ since it had an overlapping cohort with 3 of the remaining studies^28,29,47^. Half of these studies used [^11^C]NMB PET^28,29,47^, a DD2R-specific tracer, and the remaining used [^11^C]raclopride^23,27^ or [^18^F]fallypride^30^, a tracer preferring the high-affinity state of D2/3R^22^. With one exception^23^, these studies included both female and male participants, and they had a wider BMI range, with the obesity groups comprising participants with obesity from classes I to III. Only two studies (14.3%)^31,32^ reported increased DD2lR availability in the obesity group. Both included males and females and comprised, on average, participants with obesity class II. These studies used PET performed either with [^18^F]fallypride^31^ or [^11^C]PHNO PET^32^, a D3-preferring tracer^22^. All the studies attempted to minimize confounding variables, namely by excluding participants with current or past major psychiatric disorders (including eating disorders, alcohol or substance abuse), severe medical conditions (including neurological conditions and diabetes mellitus type 1 or 2), and current or past exposure to dopaminergic or anti-diabetic medication. Not all studies reported if head trauma was an exclusion factor^23,31,45,47,49^.

Regarding the four prospective cohort studies eligible for qualitative analysis^33,36,48,49^, two had partially overlapping cohorts^48,49^, leading to the decision to retain only the study with longer follow-up^49^. Of the three resulting studies, two reported increased striatal DD2lR after surgery^36,49^, one of which conducted in Europe (The Netherlands) with [^123^I]IBZM—SPECT^49^ and the other in the US using [^11^C]raclopride—PET^36^. Patients in these two studies had similar BMIs before undergoing bariatric surgery, but the mean age at inclusion differed between the two, as did follow-up, that was 6 weeks after surgery for one study^36^ and up to 3.6 years for the other^49^. The third prospective study showed decreased DD2lR after surgery^33^. This study was conducted in the US with [^11^C]raclopride—PET, had a short follow-up of approximately 7 weeks) and also included patients undergoing sleeve gastrectomy^33^, while the two studies reporting increased striatal DD2lR after surgery^36,49^ only included gastric bypass patients.

### Meta-analysis

#### Striatal dopamine D2 receptor availability in obesity

**Figure 2:**
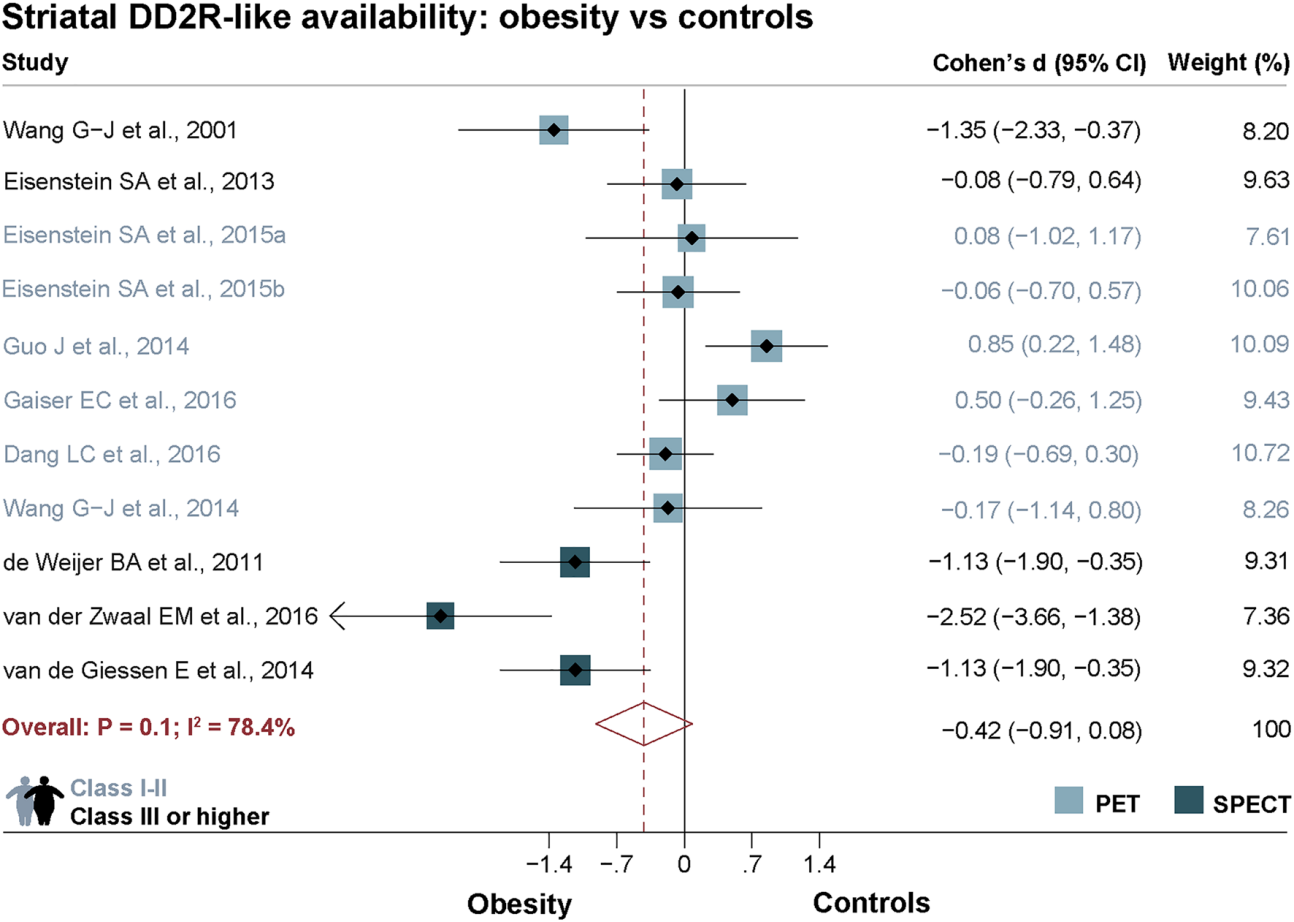
Striatal dopamine D2-like receptor availability random-effects meta-analysis in obesity and healthy controls. A pooled meta-analysis of PET and SPECT studies showed that the availability of striatal dopamine D2-like receptor (DD2lR) did not differ between the obesity and control groups. Notes: Studies are organized according to scanner type—positron emission tomography (PET—lighter blue) or single-photon emission computed tomography (SPECT—darker blue). Within scanner type, studies are ranked from highest to lowest mean body mass index (BMI) of the obesity group. Studies in black writing comprise obesity groups with class III obesity or higher, while those in grey/blue comprise class I—II obesity.

In the meta-analysis, including PET and SPECT studies, striatal DD2lR availability did not differ significantly between individuals with obesity and non-obese controls (Cohen's *d* = − 0.4, *P* = 0.1; N = 11), as shown in Fig. 2. Yet, there was significant heterogeneity across studies (I^2^ = 78.4%, *P* ≤ 0.0001). In addition, a visual assessment of the funnel plot revealed an apparent asymmetry, even though Egger's test for publication bias was not statistically significant (*P* = 0.1; Supplementary Fig. 1. Notably, in this primary analysis, differences between obesity and control groups in striatal DD2lR availability were sensitive to excluding single studies (see Supplementary Fig. 1).

**Figure 3:**
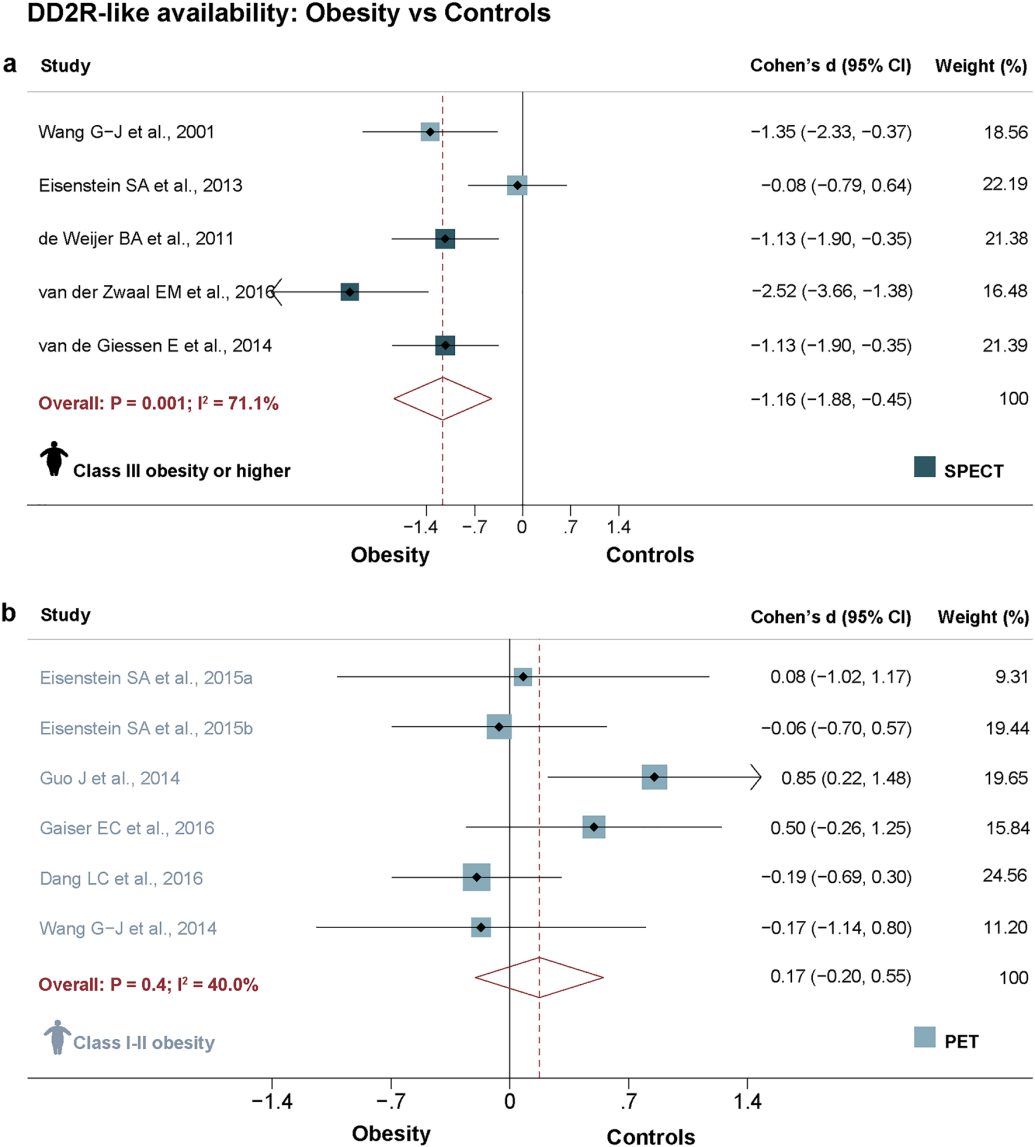
Striatal dopamine D2-like receptor availability random-effects meta-analysis in obesity and healthy controls according to classes of obesity. Meta-analysis of striatal dopamine D2-like receptor (DD2lR) availability in individuals with obesity and controls showed significant group differences favoring lower striatal DD2lR in the obesity group when only studies comprising class III obesity or higher were analyzed (Fig. 3a). No differences were found in a meta-analysis of studies that included class I-II obesity (Fig. 3b). Notes: Studies are organized according to scanner type—positron emission tomography (PET—lighter blue) or single-photon emission computed tomography (SPECT—darker blue). Within scanner type, studies range from the highest to lowest mean body mass index (BMI) of the obesity group. Class III obesity or higher: BMI ≥ 40 kg/m^2^; Class I-II obesity: BMI from 30 to < 40 kg/m^2^.

**Figure 4:**
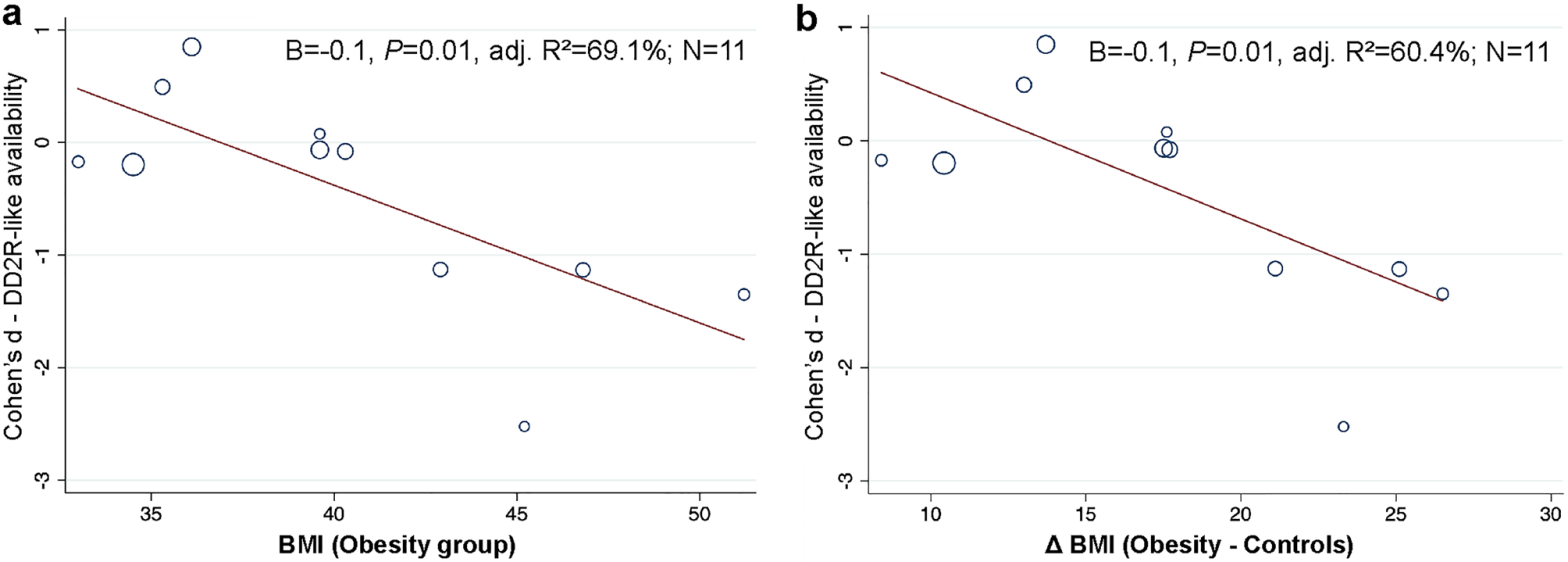
Univariate meta-regression of the association between striatal dopamine D2-like receptor availability and obesity level. An increasing body mass index (BMI) of the obesity group was significantly associated with striatal dopamine D2-like (DD2lR) availability favoring lower values in the obesity group (Fig. 4**a**). Accordingly, a higher BMI difference between the obesity and control groups (ΔBMI) was significantly associated with DD2lR availability, favoring lower values in the obesity group (Fig. 4**b**).

Some studies included patients with very high BMIs, while others comprised lower BMI ranges, so we compared striatal DD2lR availability according to the obesity level. We found lower striatal DD2lR in the obesity groups when the meta-analysis was restricted to studies comprising class III obesity or higher (Cohen's *d* = − 1.2, *P* = 0.001; N = 5, Fig. 3a). However, we did not find significant differences for studies including only class I-II obesity (Fig. 3b). We further explored the effect of obesity levels using univariate meta-regression. As shown in Fig. 4a, higher mean BMI in the obesity group was significantly associated with larger effect sizes of case–control difference in striatal DD2lR availability (B = − 0.1, *P* = 0.01, adjusted R^2^ = 69.1%; N = 11), an effect that was not found for BMI in the control group (B = 0.04, *P* = 0.9, adjusted R^2^ = − 15.0%; N = 11). Accordingly, higher ΔBMI (obesity-controls) was also significantly associated with larger effect sizes of case–control difference in DD2R availability (B = − 0.1, *P* = 0.01, adjusted R^2^ = 60.4%; N = 11; Fig. 4b).

Since age and gender could also influence striatal DD2lR availability, we tested these variables in other univariate meta-regressions. We did not find significant associations with effect sizes of case–control difference in DD2R availability for mean participant age, in either the obesity groups (B = − 0.04, *P* = 0.4, adjusted R^2^ = − 5.2%; N = 11) or the control groups (B = − 0.04, *P* = 0.3, adjusted R^2^ = 4.2%; N = 11), nor for gender distribution, in either the obesity groups (B = − 0.02, *P* = 0.1, adjusted R^2^ = 38.9%; N = 11) or the control groups (B = 0.02, *P* = 0.1, adjusted R^2^ = 31.2%; N = 11).

In further subgroup analyses, we did not find significant differences between cases and controls in meta-analyses for specific striatal regions, namely putamen, caudate, and NAc (Supplementary Fig. 3a–c). Although there were no group differences in a meta-analysis of PET studies, for SPECT studies, DD2lR availability was significantly lower in the obesity group (Cohen's *d* = − 1.5, *P* ≤ 0.0001; N = 3; Supplementary Fig. 4a, b). Consistently, there were no group differences for the majority of the radiopharmaceuticals, namely, [^11^C]raclopride (Cohen’s *d* = − 0.8, *P* = 0.2; N = 2), [^18^F]fallypride (Cohen’s *d* = 0.3, *P* = 0.5; N = 2) or [^11^C]NMB (Cohen’s *d* = − 0.1, *P* = 0.8; N = 2), but a significant difference was found for [^123^I]IBZM (Cohen’s *d* = − 1.5, *P* ≤ 0.0001; N = 3), that is the radiopharmaceuticals used for SPECT. Of note, all [^123^I]IBZM SPECT studies included class III or higher participants, while in PET studies, there was a wider BMI range. Finally, we did not find group differences in separate meta-analyses according to MRI co-registration within PET studies (Supplementary Fig. 5a,b) or study quality scores (Supplementary Fig. 6a,b).

#### Striatal Dopamine D2 Receptor availability following bariatric surgery

**Figure 5:**
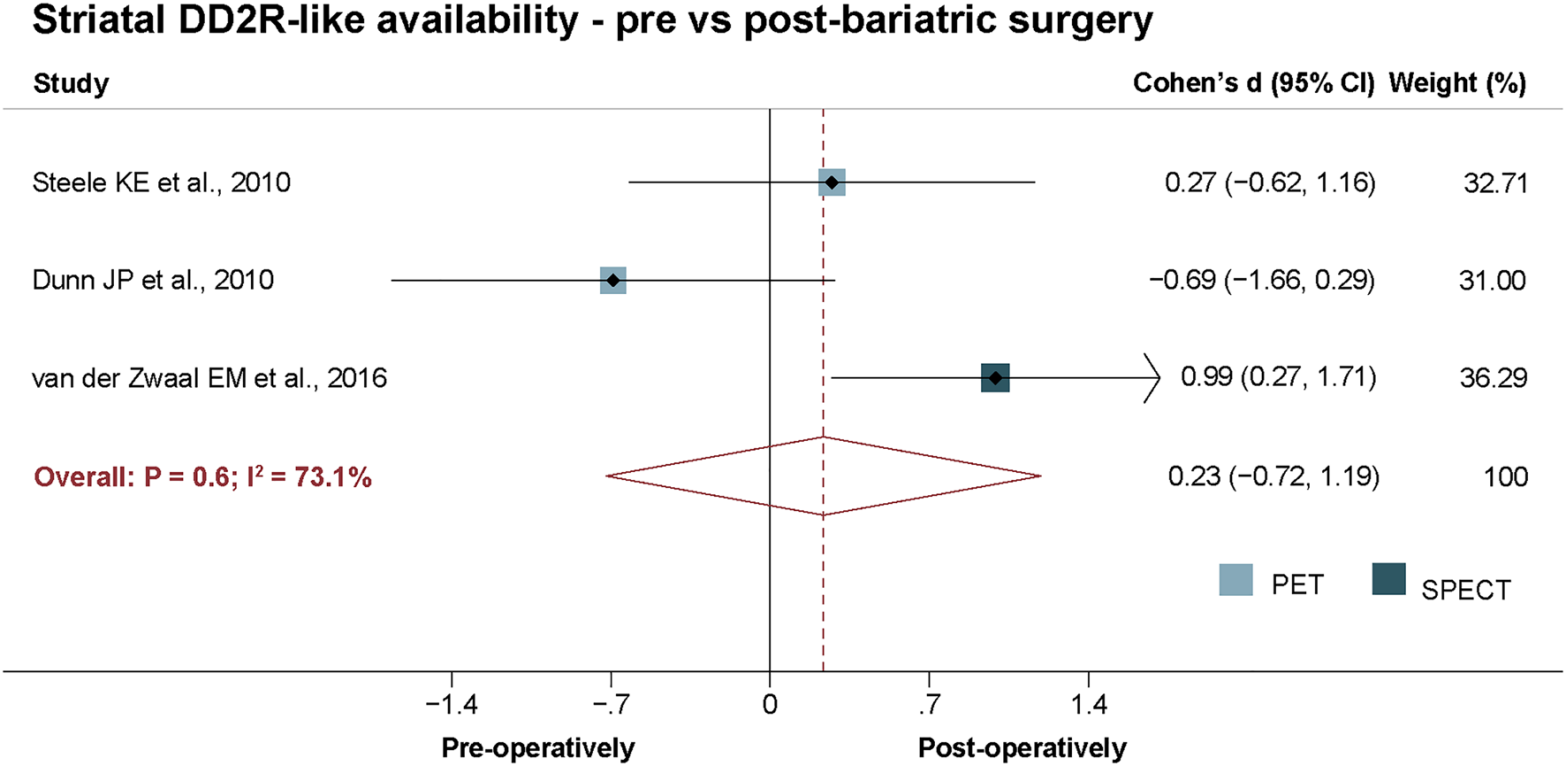
Striatal dopamine D2-like receptor availability random-effects meta-analysis after bariatric surgery. This analysis did not show changes in striatal DD2lR availability from pre- to post-bariatric surgery.

One of the primary aims of this study was to determine if striatal DD2lR availability changed after bariatric surgery. However, a small number of studies were available (N = 4)^33,36,48,49^, one of which was not included in the meta-analysis^48^ due to an overlapping sample^49^. Overall, we did not find significant changes in striatal DD2lR availability from pre- to post-surgery (Cohen's *d* = 0.2, *P* = 0.6; N = 3; Fig. 5).

## Discussion

Initial theories about reward processing in obesity suggested that lower striatal DD2lR availability was associated with a reward deficiency, contributing to overeating^10^. However, literature in this field has been challenging to reconcile given the inconsistent findings that have been reported since Wang et al.^17^, that may be explained by a nonlinear relationship between obesity and central dopaminergic physiology. Furthermore, in line with the original theory, there is some evidence that bariatric surgery may result in a normalization (i.e., increase) of DD2lR availability. Thus, this study systematically reviewed and meta-analyzed published PET and SPECT studies, comparing DD2lR availability in obesity and controls and performing prospective assessments following bariatric surgery.

In what regards to DD2lR availability in obesity relative to non-obese individuals, we did not find differences between the obesity and control groups in a pooled analysis of PET and SPECT studies. However, group differences were significant when the analysis was restricted to studies comprising class III obesity or higher. Meta-regression corroborated this finding showing inverse associations between the BMI of the obesity group or ΔBMI (obesity-controls) and the effect of obesity on DD2lR availability. Although we did not find significant changes in DD2lR availability following bariatric surgery, this finding should be cautioned given the small number of studies included. Overall, our results corroborate decreased DD2lR availability in severe obesity, although further studies are needed to determine the effect of bariatric surgery on dopaminergic physiology.

Previous literature supports our results of lower DD2lR availability in more severe forms of obesity. For instance, Van Galen et al.^22^ explained inconsistencies in these results through an inverted U-shaped association between DD2lR availability and BMI. Specifically, there would be an initial increase in DD2lR availability at moderately high BMI, particularly of high-affinity state D2/3 receptors, followed by a decrease of DD2lR availability in severe obesity^22^. Also, Horstmann A et al.^24^, suggested a non-linear (quadratic) association between changes in DD2lR availability and degree of obesity, to reconcile conflicting results. Similar non-linear associations have also been described for other reward-related measures and BMI, such as hedonic hunger and sensitivity to reward^50,51^.

Pre-clinical evidence corroborates decreased DD2lR availability in obesity. For example, mice with diet-induced obesity (DIO) had lower DD2R, but not DD1R binding relative to lean mice, associated with impaired physical activity^52^. Another study with rodent models of DIO^53^ showed decreased striatal DD2R availability and greater extracellular dopamine in obesity-prone (OP) compared with obesity-resistant (OR) rats^53^. While this study also showed lower expression of the presynaptic dopamine transporter (DAT) in OPs^53^, there seems to be no reliable association between DAT availability and obesity in clinical studies^54–56^.

The lack of group differences in the pooled analysis of PET and SPECT studies aligns with previous non-systematic reviews in this field^12,22,24^. Therefore, we decided to assess group differences, including both PET and SPECT studies, since they have comparable outcomes. However, since SPECT has a lower spatial resolution than PET, it was crucial to analyze the techniques separately and consider the effects of different radiopharmaceuticals. We found group differences in DD2lR availability in SPECT, but not PET studies, in which high BMI may have a greater influence on the results in the obesity groups than the techniques per se. It should be noted that, although we did not find significant associations between effect sizes of case–control differences and gender distribution in the obesity and the control groups, all [^123^I]IBZM SPECT studies excluded male participants. Despite differences in sensitivity and resolution in favor of PET^57^, SPECT is more widely available and the same radiopharmaceutical for DD2lR assessment has been usually used. On the other hand, there are several radiopharmaceuticals in PET, introducing variability. In the PET studies included in the meta-analysis, several used [^11^C]NMB PET^28,29,47^, all of which revealed unaltered DD2lR between groups. Contrary to radiopharmaceuticals used in the remaining studies, that also have affinity for D3 receptors, [^11^C]NMB selectively targets D2 receptors, and is not sensitive to competition from endogenous dopamine. A lack of group differences was also found in another PET study^30^ conducted with [^18^F]fallypride, suggested to have high affinity for D2/3R^22^. The same radiopharmaceutical showed increased DD2lR in the obesity group in a case–control study^31^ with a much smaller control group. The only study that used [^11^C]PHNO PET^32^, which is a D3-preferring DD2lR agonist tracer, also showed increased DD2lR in obesity relative to controls. In fact, it can be hypothesized that there could be fewer low-affinity state D2R and relatively more high-affinity states in obesity. Alternatively, there might be more striatal D3R^12^. However, considering the lower expression of D3R compared to D2Rs, it is unlikely that altered D3R availability is a determinant of group differences^58^. Indeed, some authors have suggested that the sensitivity of each radiopharmaceutical to receptor-binding competition by endogenous dopamine may contribute to group differences^22,24^. However, our results do not reveal particularly insightful differences in outcomes according to the use of both competition-sensitive^27,30^ and non-sensitive^28,29,47^ radiopharmaceuticals. Thus, lower DD2lR availability in obesity may reflect decreased availability of the receptor and/or increased endogenous synaptic dopamine levels resulting from more dopamine release^12,22^.

Research on altered endogenous synaptic dopamine levels in obesity remains inconclusive. Some have suggested that this question can be addressed with dopamine depletion studies using alpha-methyl-para-tyrosine to decrease dopamine synthesis^59^. Others have used a stimulus (e.g., food stimuli) or a pharmacological agent (e.g., dexamphetamine) to induce dopamine release and used the level of radiopharmaceutical displacement by endogenous dopamine as a proxy of dopamine release^12^. One study compared normal-weight controls with overweight or up to class II obesity patients using [^11^C]raclopride PET after a stimulus (intravenous—i.v. glucose injection) or placebo (i.v. saline NaCl 0.9%)^60^. Despite no differences between overweight and controls, there was a gender effect, with men showing decreased DD2lR binding following i.v. glucose injection (i.e., increased dopamine release), while women had the opposite result^60^. In addition, an [^123^I]IBZM SPECT study with a dexamphetamine challenge showed significant dopamine release in normal-weight controls but not in individuals with obesity, suggesting blunted dopamine release in the latter^45^. This study included, on average, class III obesity^45^, further supporting that altered striatal dopamine homeostasis may be more relevant in more severe forms of obesity.

With this study we also aimed to determine if DD2lR availability would change following bariatric surgery. However, the number of studies available was extremely small^33,36,49^, and patients were followed for a short time^33,36^. Two studies had a follow-up of six to seven weeks^33,36^, and only one study had a follow-up of at least two years^49^. In patients treated with gastric bypass surgery, the latter study found a significant increase in DD2lR availability using SPECT, which was not found in a shorter follow-up (six weeks) of the same cohort^48^. Unfortunately, such positive results have not yet been replicated, and further research is warranted to confirm or otherwise refute the effects of bariatric surgery on DD2lR availability. Also, despite one study^33^ including both gastric bypass (N = 4) and sleeve gastrectomy (N = 1), the number of patients in each group was insufficient to compare surgery types. Thus, the long-term assessment of DD2lR availability should address comparisons between gastric bypass and sleeve gastrectomy.

Results of meta-regression corroborate that altered dopaminergic physiology may be of particular relevance for more severe forms of obesity, emphasizing that within obesity there is biological and phenotypical heterogeneity. Beyond differences at the BMI level, obesity is inherently multifactorial, and can be differently expressed across individuals in terms of metabolism^61^ and psycho-biological variables such as reward-related taste sensitivity^62,63^, addiction-like feeding behaviors^63,64^, hedonic hunger^50,64^, eating style^63^, and reward sensitivity^65^. In the present study, the categorization of obesity solely based on BMI provided an insight on a subgroup that may be more vulnerable to changes in dopaminergic physiology, i.e., severe obesity. Among theories that have explained individuals differences in food reward sensitivity, the *dynamic vulnerability model of obesity*^66^ proposes that an initial heightened responsivity to palatable foods promotes overeating of these foods, but that upon repeated consumption of palatable foods dopamine signaling may decrease, reducing responsivity to palatable food cues over time^66^. This model thus assumes a baseline level of reward sensitivity, including to palatable foods, that is modulated by the level of dietary exposure to palatable foods over time^66^. In light of this model, we can hypothesize that individuals with severe obesity are more likely to present early biological changes that heighten reward sensitivity, which is then aggravated by a more frequent or longer exposure to consumption of palatable foods.

Although our findings suggest that central dopaminergic changes are more relevant for higher obesity classes, it is unlikely that obesity, and even severe obesity, can be translated into a single dopamine-related determinant. Within the dopamine system, nuclear medicine studies have typically targeted a single substrate, cross-sectionally, in small samples^67^. Nevertheless, this approach is not in line with the intricacy of obesity nor of dopaminergic physiology^67^. Hence, for personalized treatment of obesity, it will be fundamental to characterize dopamine profiles within obesity subtypes and determine their functional effects. Indeed, we propose that future PET/SPECT studies should aim to characterize and select participants in further detail, including metabolically and behaviorally. Additionally, multi-radiopharmaceutical studies to assess, for example, striatal receptor availability and dopamine synthesis capacity in the same individuals, ideally in larger samples with a wider BMI range, can provide useful information^67^. Despite the obvious gaps that exist in the field, the results reported here corroborate brain changes in obesity, that may contribute to behavioral factors such as poor compliance to diet and exercise. Indeed, if this is accurate, it is possible that pharmacologic options targeting the dopamine system may be useful for treatment of obesity as co-adjuvants of behavioral changes. Lastly, given that GLP-1 analogues may also have effects on human reward-related feeding behavior^68^, studies addressing the effects of GLP-1 analogues on central dopamine function may be an interesting avenue for future studies.

The interpretation of this study requires an understanding of its limitations. First, the weighted mean computed as a proxy for striatal DD2lR availability is not as accurate as a direct measure of striatal BP. Second, our meta-analyses included a relatively low number of studies and sample sizes. For example, the primary meta-analysis assessing DD2lR availability in obesity and control groups had 11 studies, close to the minimal number of recommended studies. While one study included more than 100 participants^30^, it did not have a pure case–control design but was instead a cohort with a wide BMI range, including an obesity group of 13.4% of the total sample. Importantly, in the primary analysis, despite publication bias not being significant according to Egger's test, the funnel plot had some asymmetry. Specifically, there is a lack of large studies showing high negative effect estimates (i.e., lower DD2lR availability in obesity vs. controls). Indeed, several factors, including inclusion criteria or challenging methods for large-scale applications, can lead smaller studies to find more significant effects, resulting in asymmetry^69^. These challenges range from the cost and half-life of radiopharmaceuticals to scan time and exclusion criteria in nuclear medicine imaging. The primary meta-analysis also had substantial methodological heterogeneity, expected when pooling different scanner types and radiopharmaceuticals. The comparison between obesity and control groups in striatal DD2lR availability was sensitive to the exclusion of single studies, such as Guo J et al.^31^, the only study showing lower striatal DD2R availability in controls. While heterogeneity was an important challenge for our analyses, we note that, regarding our search protocol, we minimized database bias by using three reference databases and screening each chosen article reference list for articles missed by our syntax.

## Conclusions

This study was the first systematic review and meta-analysis to assess DD2lR availability in obesity, including PET and SPECT studies and its prospective assessment following bariatric surgery. DD2lR availability may not significantly differ between individuals with obesity and non-obesity controls across all obesity ranges. However, DD2lR availability seems to be lower in severe obesity. On the other hand, there does not appear to be normalization of DD2lR availability in the short term after bariatric surgery, which will require additional studies with longer follow-ups and comparisons between gastric bypass and sleeve gastrectomy. Overall, the analysis herein reported reinforces the importance of studying determinants of altered dopaminergic physiology in obesity and potential contributions to obesity phenotypes and variability in treatment response. This advance in knowledge will contribute to precision medicine in treating patients with obesity.

## Supplementary Information

Supplementary Information 1.

Supplementary Information 2.

## Data Availability

The datasets generated during and/or analyzed during the current study are available from the corresponding author upon reasonable request.
