# Supplementary material for: Striatal dopamine D2-like receptors availability in obesity and its modulation by bariatric surgery: a systematic review and meta-analysis
Source: Sci Rep. 2023 Mar 27;13:4959. doi: 10.1038/s41598-023-31250-2 (PMC10042861; doi:10.1038/s41598-023-31250-2)
Supplement: Supplementary file 2 — Supplementary Information 2. [file 41598_2023_31250_MOESM2_ESM.pdf]

Striatal dopamine D2-like receptors availability in obesity and its modulation by bariatric surgery: a systematic review and meta-analysis.

Gabriela Ribeiro, Ana Maia, Gonçalo Cotovio, Francisco P.M. Oliveira, Durval C. Costa, Albino J. Oliveira-Maia.

Supplementary Material

1. **Supplementary Tables**

   Supplementary Table 1. Search syntax.

   Supplementary Table 2. Methodological characteristics of eligible studies.

   Supplementary Table 3. Summary of critical appraisal of included studies using the Newcastle-Ottawa scale for case-control or cohort studies.

2. **Supplementary Figures**

   Supplementary Figure 1. Bias assessment of striatal dopamine D2-like receptor availability random-effects meta-analysis in obesity and healthy controls.

   Supplementary Figure 2. Leave-one-out meta-analysis of striatal dopamine D2-like receptor availability in obesity and healthy controls.

   Supplementary Figure 3. Striatal dopamine D2-like receptor availability random-effects meta-analysis in obesity and healthy controls within striatal sub-regions.

   Supplementary Figure 4. Striatal dopamine D2-like receptor availability random-effects meta-analysis in obesity and healthy controls according to scan type.

   Supplementary Figure 5. Striatal dopamine D2-like receptor availability random-effects meta-analysis in obesity and healthy controls according to PET studies with or without MRI co-registration.

   Supplementary Figure 6. Striatal dopamine D2-like receptor availability random-effects meta-analysis in obesity and healthy controls according to quality of the studies assessed by the Newcastle-Ottawa scale.

3. **Supplementary References**

Supplementary Table 1. Search syntax.

Bibliographic databases	Nuclear medicine imaging	Striatal dopamine D2 receptors availability	Obesity	Bariatric surgery
PubMed[a]<br>Web of Science[a]<br>Embase[a]	(1) ‘Positron Emission Tomography’;<br>(2) ‘PET’<br>(3) ‘Single Photon Emission Computed Tomography’<br>(4) ‘SPECT’	(5) ‘dopamine’<br>(6) ‘striatum’<br>(7) ‘basal ganglia’<br>(8) ‘putamen’<br>(9) ‘caudate’<br>(10) ‘accumbens’<br>(11) ‘D2’<br>(12) ‘DD2R’	(13) ‘obesity’<br>(14) ‘overweight’<br>(15) ‘body mass index’<br>(16) ‘BMI’	(17) ‘bariatric’<br>(18) ‘gastric bypass’<br>(19) ‘sleeve’<br>(20) ‘gastrectomy’
	The Boolean terms were: [(1) OR (2) OR (3) OR (4)] AND [(5) OR (6) OR (7) OR (8) OR (9) OR (10) OR (11) OR (12)] AND [(13) OR (14) OR (15) OR (16) OR (17) OR (18) OR (19) OR (20)].			

[a]The following filters were applied: Humans; English, Portuguese, Spanish, French, and German.

Supplementary Table 2. Methodological characteristics of eligible studies.

Publication	Country of Publication	Radiotracer				Scan type	MRI	Regions of interest	Reference region	Acquisition time
		Reference	Affinity[a]		Sensitivity to endogenous competition[a]					
			D2	D3						
**Case-control studies**										
Wang G-J et al., 2001	US	[^{11}C]raclopride	++	++	+	PET	✗	Striatum	Cerebellum	60 min
Volkow ND et al., 2008	US	[^{11}C]raclopride	++	++	+	PET	✗	Striatum	Cerebellum	60 min
de Weijer BA et al., 2011	NL	[^{123}I]IBZM	+	+	+	SPECT	✗	Striatum	Occipital	60 min, 120 min PI
van De Giessen E et al., 2014	NL	[^{123}I]IBZM	+	+	+	SPECT	✗	Striatum	Occipital	300 min
Karlsson HK et al., 2015	FI	[^{11}C]raclopride	++	++	+	PET	✓	Put, d-caud, VS	Cerebellum	51 min
Pepino MY et al., 2016	US	[^{11}C]NMB	++	-	-	PET	✓	Put, caud, NAc	Cerebellum	2 h
Eisenstein SA et al., 2013	US	[^{11}C]NMB	++	-	-	PET	✓	Put, caud, NAc[b]	Cerebellum	2 h
Eisenstein SA et al., 2015a	US	[^{11}C]NMB	++	-	-	PET	✓	Put, caud, NAc[b]	Cerebellum	2 h
Eisenstein SA et al., 2015b	US	[^{11}C]NMB	++	-	-	PET	✓	Striatum	Cerebellum	2 h
Guo J et al., 2014	US	[^{18}F]fallypride	+++	+++	+	PET	✓	Put, caud, NAc[b]	Cerebellum	3.5 h, 2 breaks 10 min
Gaiser EC et al., 2016	US	[^{11}C]PHNO	++	++(+)	++	PET	✓	Put, caud, VS[b]	Cerebellum	120 min
Dang LC et al., 2016	US	[^{18}F]fallypride	+++	+++	+	PET	✓	Put, caud NAc[b]	Cerebellum	3.5 h, 2 breaks 15 min
Wang G-J et al., 2014	US	[^{11}C]raclopride	++	++	+	PET	✗	Put, caud, NAc[b]	Cerebellum	60 min
**Prospective cohort studies**										
de Weijer BA et al., 2014	NL	[^{123}I]IBZM	+	+	+	SPECT	✗	Striatum	Occipital	60 min, 120 min PI
van der Zwaal EM et al., 2016	NL	[^{123}I]IBZM	+	+	+	SPECT	✗	Striatum	Occipital	60 min, 120 min PI

Steele KE et al., 2010	US	[11C]raclopride	++	++	+	PET	✓	Striatum	Cerebellum	90 min
Dunn JP et al., 2010	US	[11C]raclopride	++	++	+	PET	✓	Put, caud, VS[b]	Cerebellum	3.5 h, 2 breaks 15 min

Note: Studies are organized by the body mass index level of the obesity group.

Abbreviations: US – United States; NL - The Netherlands; FI – Finland; [123I] IBZM - Iodobenzamide; [11C] NMB - N-[11C] methyl) benperidol; [11C] PHNO - [11C]-4-propyl-3,4,4a,5,6,10b-hexahydro-2H-naphtho[1,2-b] [1,4] oxazin-9-ol hydrochloride. PET - Positron emission tomography; SPECT - Single-photon emission computed tomography; Put - putamen; Caud - caudate; d-caudate - dorsal caudate; VS - ventral striatum; NAc - Nucleus accumbens.

[a]The classification based on van Galen KA et al., 2018[1], ranges from irrelevant (-) to very high (+++).

[b]A weighted mean using the binding potential of these regions of interest was performed to estimate striatal dopamine D2-like receptor availability.

Supplementary Table 3. Summary of critical appraisal of included studies using the Newcastle-Ottawa scale for case-control or cohort studies.

**Reference**	**Selection (max 4 stars)**	**Comparability (max 2 stars)**	**Exposure/Outcome (max 3 stars)**	**Total (max 9 stars)**
**Case-control studies**				
Wang G-J et al., 2001	**		***	*****
Volkow ND et al., 2008	**		***	*****
de Weijer BA et al., 2011	**	**	**	******
van de Giessen E et al., 2014	**	**	***	*******
Karlsson HK et al., 2015	**	**	***	*******
Pepino MY et al., 2016	**	**	***	*******
Eisenstein SA et al., 2013	**	**	***	*******
Eisenstein SA et al., 2015a	**	**	***	*******
Eisenstein SA et al., 2015b	**	**	***	*******
Guo J et al., 2014	**	*	***	******
Gaiser EC et al., 2016	***	**	***	********
Dang LC et al., 2016	**	**	***	*******
Wang G-J et al., 2014	*		***	****
**Prospective cohort studies**				
de Weijer BA et al., 2014	****	**	***	*********
van der Zwaal EM et al., 2016	****	**	***	*********
Dunn JP et al., 2010	****	*	***	********
Steele KE et al., 2010	****	**	***	*********
Dunn JP et al., 2010	****	*	***	********

Notes: We assessed the methodological quality of the studies included in the systematic review using the Newcastle-Ottawa Scale (NOS)[2] for case-control or cohort studies. The NOS consists of nine items grouped in three subscales: (i) selection of study groups, (ii) comparability of groups, and (iii) ascertainment of outcome (cohort studies) or (iii) exposure (case-control studies). The scores range from 0 to 9 ‘stars’ (*). We considered a study awarded a score equal to or higher than eight[3] as a high-quality study in the current work.

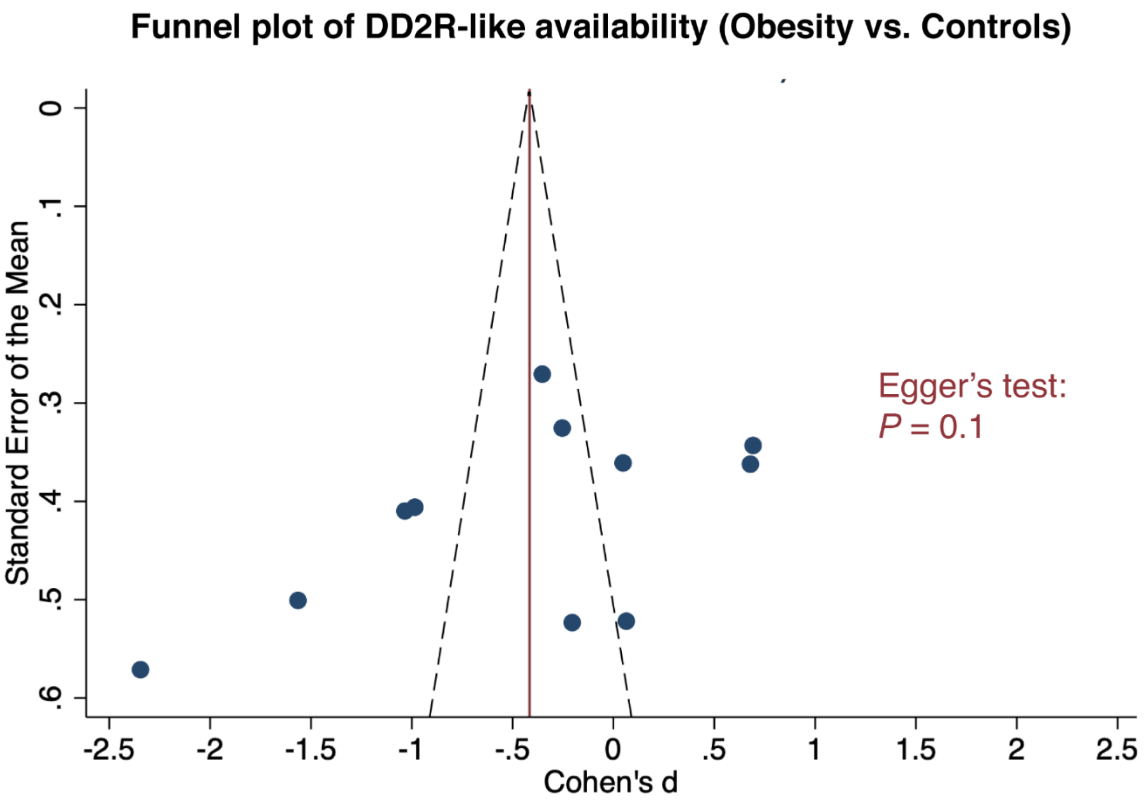

Supplementary Figure 1. Bias assessment of striatal dopamine D2-like receptor availability random effects meta-analysis in obesity and healthy controls. The effect estimate (Cohen's d) is plotted on the x-axis and a measure of study precison (or study size – standard error of the mean) is plotted in the y-axis. Visual assessment of the funnel plot shows an apparent asymmetry. However, Egger's test for publication bias was not statistically significant.

Leave-one-out meta-analysis
Striatal DD2R-like availability (Obesity vs. Controls)

Study omitted	Estimate	[95% Conf.	Interval]
1 Wang G–J et al., 2001	-.33086166	-.84171635	.17999303
2 Eisenstein SA et al., 2013	-.45790255	-1.0117207	.0959156
3 Eisenstein SA et al., 2015a	-.46000302	-.99325109	.073245
4 Eisenstein SA et al., 2015b	-.46202606	-1.0229361	.09888399
5 Guo J et al., 2014	-.54820973	-1.0216908	-.07472862
6 Gaiser EC et al., 2016	-.51135266	-1.0383592	.01565387
7 Dang LC et al., 2016	-.45226616	-1.0343634	.12983111
8 Wang G–J et al., 2014	-.44223791	-.98231971	.09784389
9 de Weijer BA et al., 2011	-.3417359	-.86157709	.17810532
10 van der Zwaal EM et al., 2016	-.24060385	-.67767298	.19646527
11 Van de Giessen E et al., 2014	-.3422341	-.86238968	.17792149
Combined	-.41591179	-.91478579	.0829622

Supplementary Figure 2. Leave-one-out meta-analysis of striatal dopamine D2-like receptor availability in obesity and healthy controls. Differences between obesity and control groups in striatal dopamine D2-like receptor (DD2lR) availability were sensitive to, the exclusion of Guo J et al., 2014, that resulted in significant group differences, as shown by the 95% confidence interval.

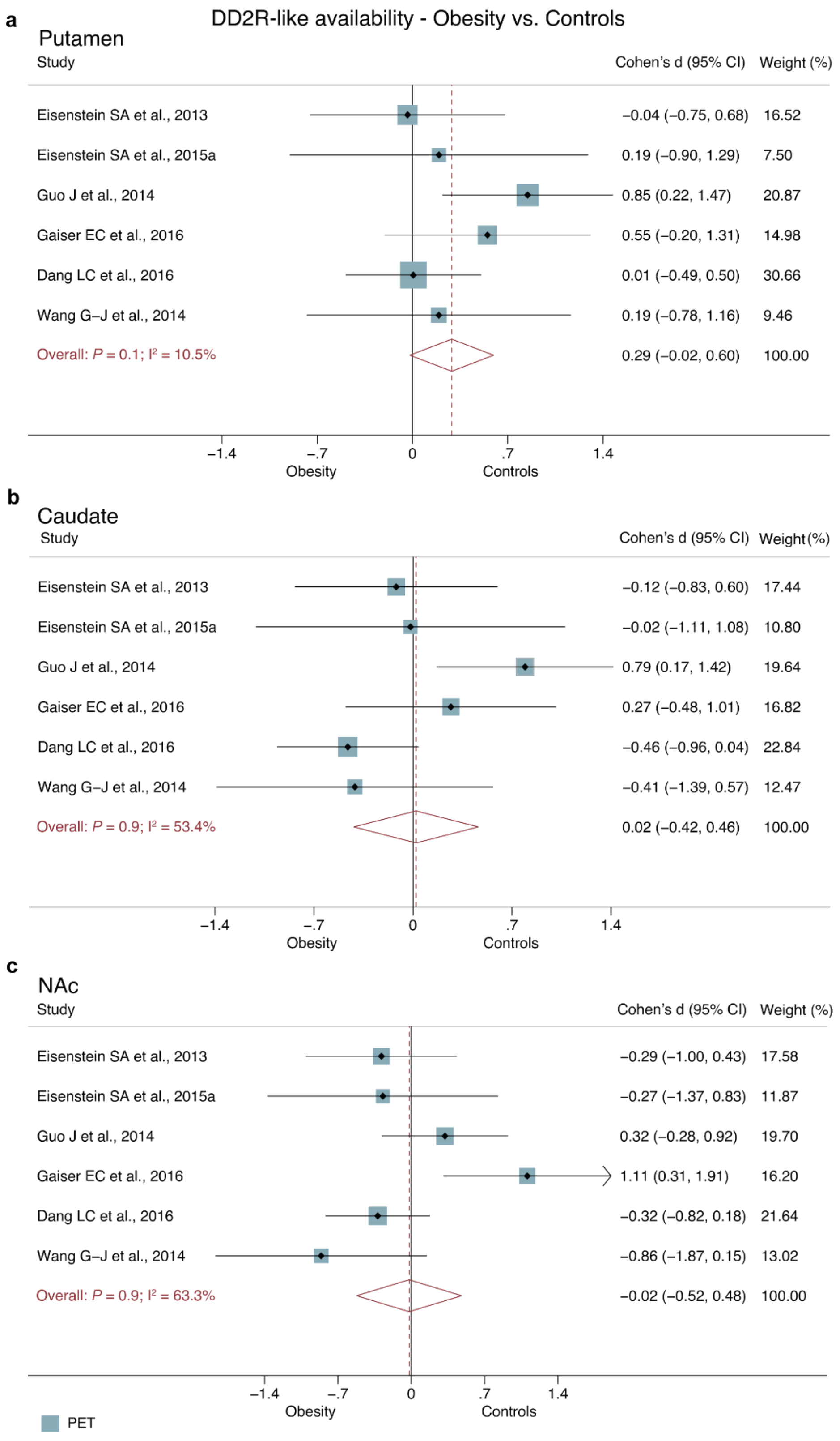

Supplementary Figure 3. Striatal dopamine D2-like receptor availability random effects meta-analysis in obesity and healthy controls within striatal sub-regions. Striatal dopamine D2-like receptor (DD2lR) availability was similar between obesity and healthy controls independently of the striatal region analyzed, namely putamen (**Supplementary Figure 3a**), caudate (**Supplementary Figure 3b**) and nucleus accumbens - NAc (**Supplementary Figure 3c**).

Notes: DD2lR availability values for striatal sub-regions were only available for a subset of the studies.

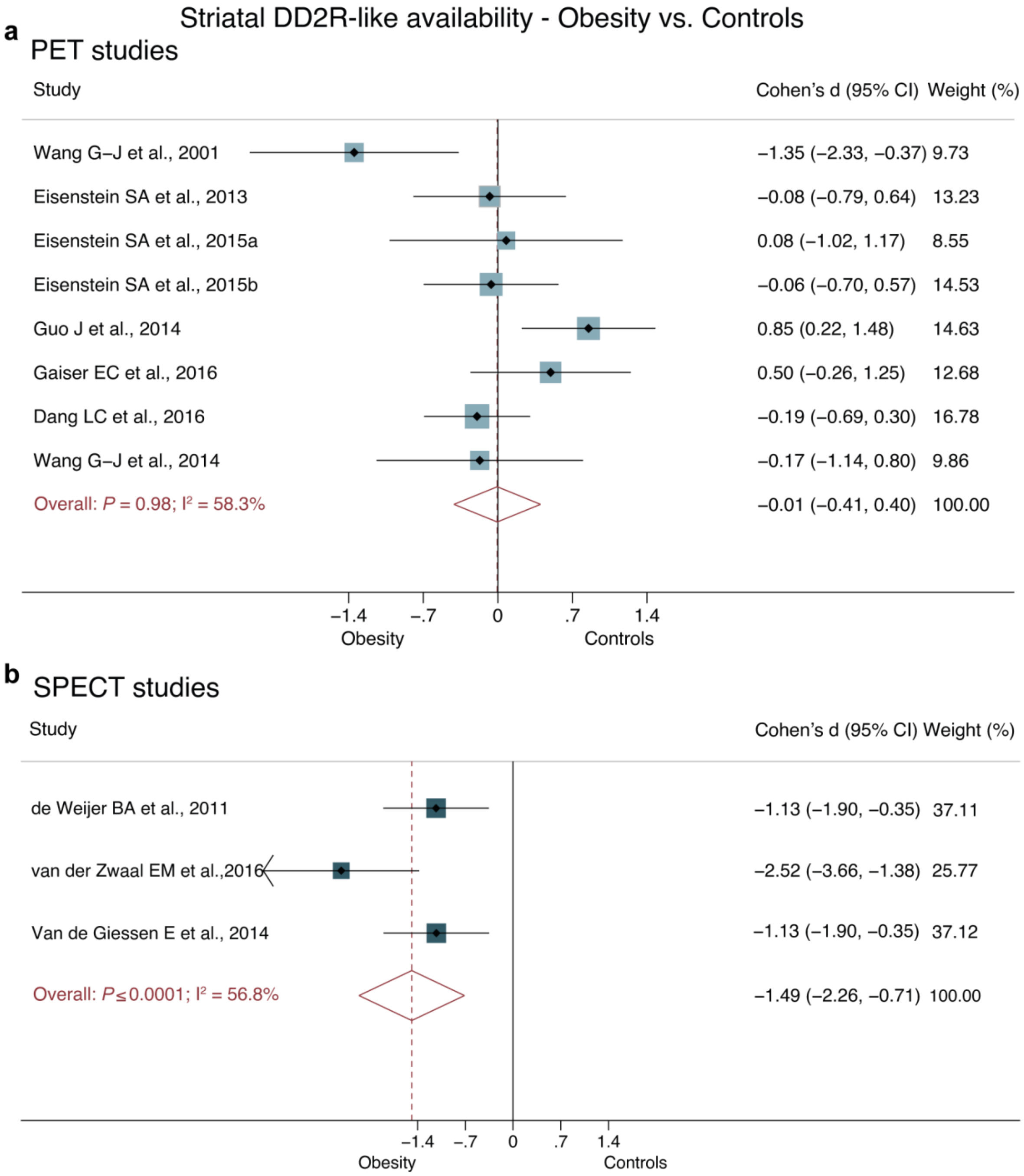

Supplementary Figure 4. Striatal dopamine D2-like receptor availability random effects meta-analysis in obesity and healthy controls according to scan type.

Striatal dopamine D2-like receptor (DD2lR) availability in obesity and healthy controls significantly differed between Positron emission tomography (PET) and Single-photon emission computed tomography (SPECT) studies. While in PET studies there were no group differences (**Supplementary Figure 4a),** in SPECT studies the groups significantly differed, favoring lower DD2R-like availability in the obesity group (**Supplementary Figure 4b)**.

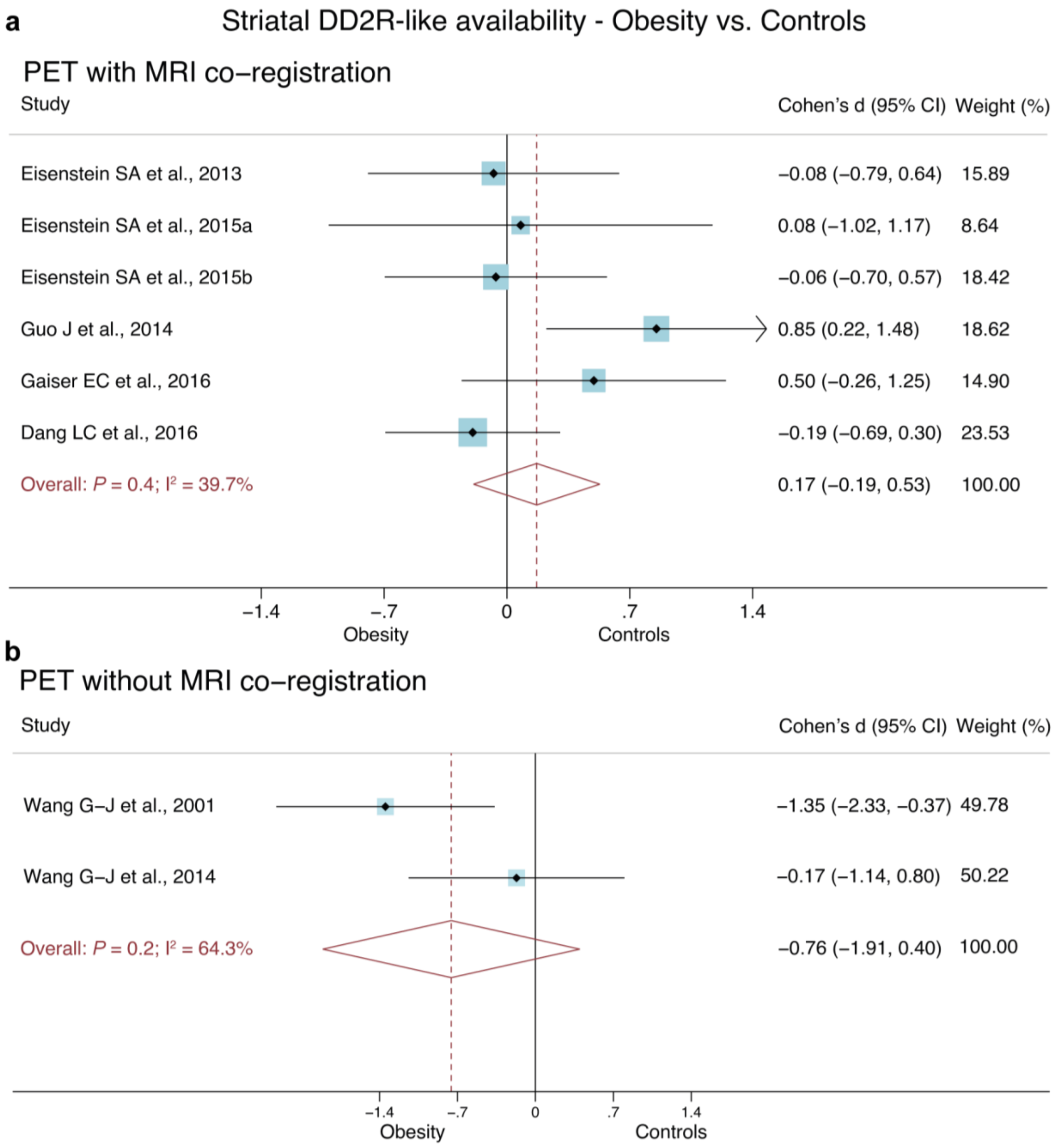

Supplementary Figure 5. Striatal dopamine D2-like receptor availability random-effects meta-analysis in obesity and healthy controls according to PET studies with or without MRI co-registration.

Within Positron emission tomography (PET) studies, striatal dopamine D2-like receptor (DD2lR) availability in obesity and healthy controls didn't differ between studies that performed Magnetic Resonance Imaging (MRI) co-registration (**Supplementary Figure 5a**) and those that did not (**Supplementary Figure 5b**).

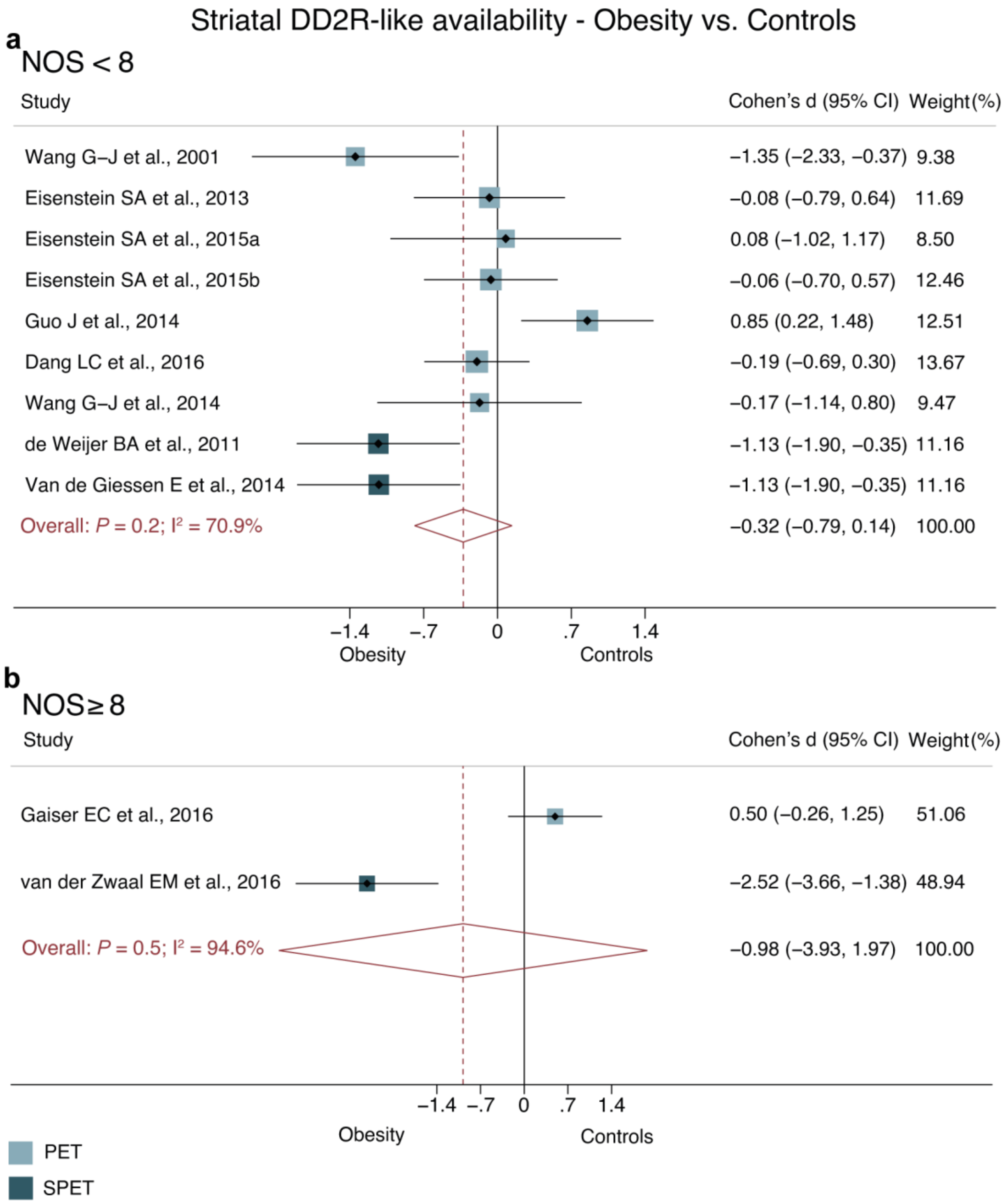

Supplementary Figure 6. Striatal dopamine D2-like receptor availability random-effects meta-analysis in obesity and healthy controls according to Newcastle-Ottawa scale scores**.**

Striatal dopamine D2-like receptor (DD2lR) availability in obesity and healthy controls didn't differ between studies with Newcastle-Ottawa scale (NOS) scores lower than 8 (**Supplementary Figure 6a**) and equal or higher than 8 (**Supplementary Figure 6b**).

Supplementary References

1. van Galen KA, ter Horst KW, Booij J, la Fleur SE, Serlie MJ. The role of central dopamine and serotonin in human obesity: lessons learned from molecular neuroimaging studies. *Metabolism*. 2018;85:325-339. doi:10.1016/j.metabol.2017.09.007

2. Peterson J, Welch V, Losos M, Tugwell P. The Newcastle-Ottawa scale (NOS) for assessing the quality of nonrandomised studies in meta-analyses. *Ott Ott Hosp Res Inst*. Published online 2011:1-12.

3. Salvi V, Grua I, Cerveri G, Mencacci C, Barone-Adesi F. The risk of new-onset diabetes in antidepressant users - A systematic review and meta-analysis. *PloS One*. 2017;12(7):e0182088. doi:10.1371/journal.pone.0182088
